# Continuous Glucose Monitoring With Low-Carbohydrate Nutritional Coaching to Improve Type 2 Diabetes Control: Randomized Quality Improvement Program

**DOI:** 10.2196/31184

**Published:** 2022-02-02

**Authors:** Dina H Griauzde, Grace Ling, Daniel Wray, Melissa DeJonckheere, Kara Mizokami Stout, Laura R Saslow, Jill Fenske, David Serlin, Spring Stonebraker, Tabassum Nisha, Colton Barry, Rodica Pop-Busui, Ananda Sen, Caroline R Richardson

**Affiliations:** 1 VA Ann Arbor Healthcare System Ann Arbor, MI United States; 2 Department of Internal Medicine Division of General Medicine University of Michigan Ann Arbor, MI United States; 3 Institute for Healthcare Policy and Innovation University of Michigan Ann Arbor, MI United States; 4 Department of Family Medicine University of Michigan Ann Arbor, MI United States; 5 Twine Clinical Consulting LLC Park City, UT United States; 6 Department of Internal Medicine Division of Metabolism, Endocrinology and Diabetes University of Michigan Ann Arbor, MI United States; 7 Department of Health Behavior and Biological Sciences School of Nursing University of Michigan Ann Arbor, MI United States; 8 Department of Biostatistics School of Public Health University of Michigan Ann Arbor, MI United States

**Keywords:** type 2 diabetes mellitus, continuous glucose monitoring, low-carbohydrate counseling

## Abstract

**Background:**

Type 2 diabetes mellitus (T2DM) is a leading cause of morbidity and mortality globally, with adverse health consequences largely related to hyperglycemia. Despite clinical practice guideline recommendations, effective pharmacotherapy, and interventions to support patients and providers, up to 60% of patients diagnosed with T2DM are estimated to have hemoglobin A_1c_ (HbA_1c_) levels above the recommended targets owing to multilevel barriers hindering optimal glycemic control.

**Objective:**

The aim of this study is to compare changes in HbA_1c_ levels among patients with suboptimally controlled T2DM who were offered the opportunity to use an intermittently viewed continuous glucose monitor and receive personalized low-carbohydrate nutrition counseling (<100 g/day) versus those who received usual care (UC).

**Methods:**

This was a 12-month, pragmatic, randomized quality improvement program. All adult patients with T2DM who received primary care at a university-affiliated primary care clinic (N=1584) were randomized to either the UC or the enhanced care (EC) group. Within each program arm, we identified individuals with HbA_1c_ >7.5% (58 mmol/mol) who were medically eligible for tighter glycemic control, and we defined these subgroups as UC–high risk (UC-HR) or EC-HR. UC-HR participants (n=197) received routine primary care. EC-HR participants (n=185) were invited to use an intermittently viewed continuous glucose monitor and receive low-carbohydrate nutrition counseling. The primary outcome was mean change in HbA_1c_ levels from baseline to 12 months using an intention-to-treat difference-in-differences analysis comparing EC-HR with UC-HR groups. We conducted follow-up semistructured interviews to understand EC-HR participant experiences with the intervention.

**Results:**

HbA_1c_ decreased by 0.41% (4.5 mmol/mol; *P*=.04) more from baseline to 12 months among participants in the EC-HR group than among those in UC-HR; however, only 61 (32.9%) of 185 EC-HR participants engaged in the program. Among the EC-HR participants who wore continuous glucose monitors (61/185, 32.9%), HbA_1c_ was 1.1% lower at 12 months compared with baseline (*P*<.001). Interviews revealed themes related to EC-HR participants’ program engagement and continuous glucose monitor use.

**Conclusions:**

Among patients with suboptimally controlled T2DM, a combined approach that includes continuous glucose monitoring and low-carbohydrate nutrition counseling can improve glycemic control compared with the standard of care.

## Introduction

### Background

Type 2 diabetes mellitus (T2DM) is a leading cause of morbidity and mortality globally, with adverse health consequences largely related to hyperglycemia [1]. Unfortunately, despite clinical practice guideline recommendations [2], effective pharmacotherapy [3], and interventions to support patients and providers [4-6], up to 60% of patients with diagnosed T2DM are estimated to have hemoglobin A_1c_ (HbA_1c_) levels above recommended targets [2,7]. Multilevel barriers hinder optimal glycemic control, including those at the level of patients (eg, medication nonadherence [8]), providers (eg, clinical inertia [9,10]), and health systems (eg, lack of support resources [11]). Such barriers may be exacerbated by the high costs of many T2DM medications, including insulin [12].

Novel strategies that can be sustained and scaled in diverse clinical settings are needed to help more patients with T2DM to achieve the dual goals of glycemic control and reduced medication burden. Growing evidence suggests that these goals are achievable through dietary carbohydrate restriction. Very low–carbohydrate and low-carbohydrate diets (defined as <10% and 10%-26% of total daily energy from carbohydrates, respectively) have been successfully used in clinical trial settings to manage and reverse T2DM [13,14]. Accordingly, clinical practice guidelines for T2DM now support the use of carbohydrate-restricted meal plans among patients with T2DM who (1) are not meeting glycemic targets, (2) wish to reduce the use of antihyperglycemic agent, or (3) prefer such a dietary approach [15,16]. However, to date, few strategies exist to support the use of carbohydrate-restricted meal plans in general practice settings, as such diets often require intensive personalized instruction and close monitoring with medication adjustments to mitigate the risk of hypoglycemia among patients treated with agents other than metformin [17-19].

A promising strategy to effectively, efficiently, and safely support the use of carbohydrate-restricted meal plans among patients with suboptimally controlled T2DM may be through continuous glucose monitoring (CGM). CGM can support patients’ self-education and self-management by providing real-time information on individuals’ glycemic responses to specific foods. CGM technology—historically used in the management of type 1 diabetes mellitus—is now less expensive, user-friendly, and increasingly used to guide medication treatment decisions among patients with T2DM [20,21]. However, little is known about the potential role of CGM technology as a tool to help patients initiate and sustain behavior changes. In a previously published pilot study of 15 patients with prediabetes, we showed that CGM plus low-carbohydrate coaching is an acceptable and feasible approach to support dietary behavior change [22].

### Objectives

We hypothesized that an intervention combining the use of CGM technology with dietitian-delivered education focused on dietary carbohydrate restriction would be effective in altering patients’ eating behaviors and improving glycemic control among patients with suboptimally controlled T2DM. The primary objective of this pragmatic randomized quality improvement (QI) program is to compare mean change in HbA_1c_ levels among patients with suboptimally controlled T2DM (defined as HbA_1c_ ≥7.5%) who were offered the opportunity to use a CGM and receive nutritional counseling versus those who received usual care (UC).

## Methods

### Study Design

We conducted a 12-month, pragmatic, randomized controlled population level QI program evaluation. Although we could have conducted a more traditional, simple 2-arm randomized controlled trial, we wanted to be able to understand the potential reach of the QI program in a typical primary care office setting. Thus, rather than randomizing participants after obtaining consent as done in a usual research study, we first identified and randomized the entire population of adult patients with T2DM seen in the clinic to either a UC or an enhanced care (EC) arm. We then attempted to contact every patient who was randomized to the EC arm who had an HbA_1c_ level >7.5% (58 mmol/mol) and for whom tighter glycemic control was medically appropriate and invited them to engage in the program. This allowed us to estimate the potential reach of the program outside of a consented research setting. All patients who engaged in the study provided clinical consent, but only a subset of those who engaged consented to allow detailed data to be used for subsequent research or education. After 1 year, those who were eligible and randomized to the UC arm were also offered the opportunity to engage in the intervention, but the data presented here do not include the waitlist control results.

The evaluation was pragmatic in that the recruitment and intervention procedures were integrated into usual clinical workflows and were conducted by clinic staff rather than by research staff. We used the Pragmatic Explanatory Continuum Indicator Summary [23] to design the study and select strategies that were more pragmatic (rather than explanatory), thus increasing the likelihood that the program could be scaled and sustained in real-world clinical settings.

Owing to the complex evaluation design, the program was reviewed under 2 separate institutional review board (IRB) applications by the University of Michigan’s IRB. The QI program was deemed exempt and not regulated and was granted a waiver of informed consent for cohort identification and participant tracking (HUM00147295). This component included the quantitative evaluation of the primary outcomes assessed through deidentified cohort summary data. The second IRB application was applied only to the data repository subcohort. All individuals who engaged in the program were invited to participate in a data repository, which allowed study team members to review their complete electronic health record (EHR) for current and subsequent research and education. Participation in the data repository was voluntary and required written informed consent (HUM00148100). The program evaluation was conducted from November 2018 to November 2019.

### Participants and Setting

This QI program took place at a university-affiliated family medicine clinic staffed by family medicine physicians, residents, and advanced practice providers. Although detailed patient-level sociodemographic data were limited owing to the QI nature of this program, most patients served by the clinic are White and have private insurance or Medicare.

Using Data Direct [24]—the Michigan Medicine web-based tool for accessing data from >4 million patients within the health system—we identified patients aged ≥21 years with T2DM, as determined by EHR-based problem list diagnosis; with HbA_1c_ ≥6.5%; or active prescription for any antihyperglycemic medication other than metformin (N=1584).

### Randomization

All individuals were randomized to one of the two program groups using 1:1 randomization with stratification based on age, gender, and BMI: UC or EC. The allocation sequence was generated using STATA 16.0 (StataCorp LLC).

**Figure 1:**
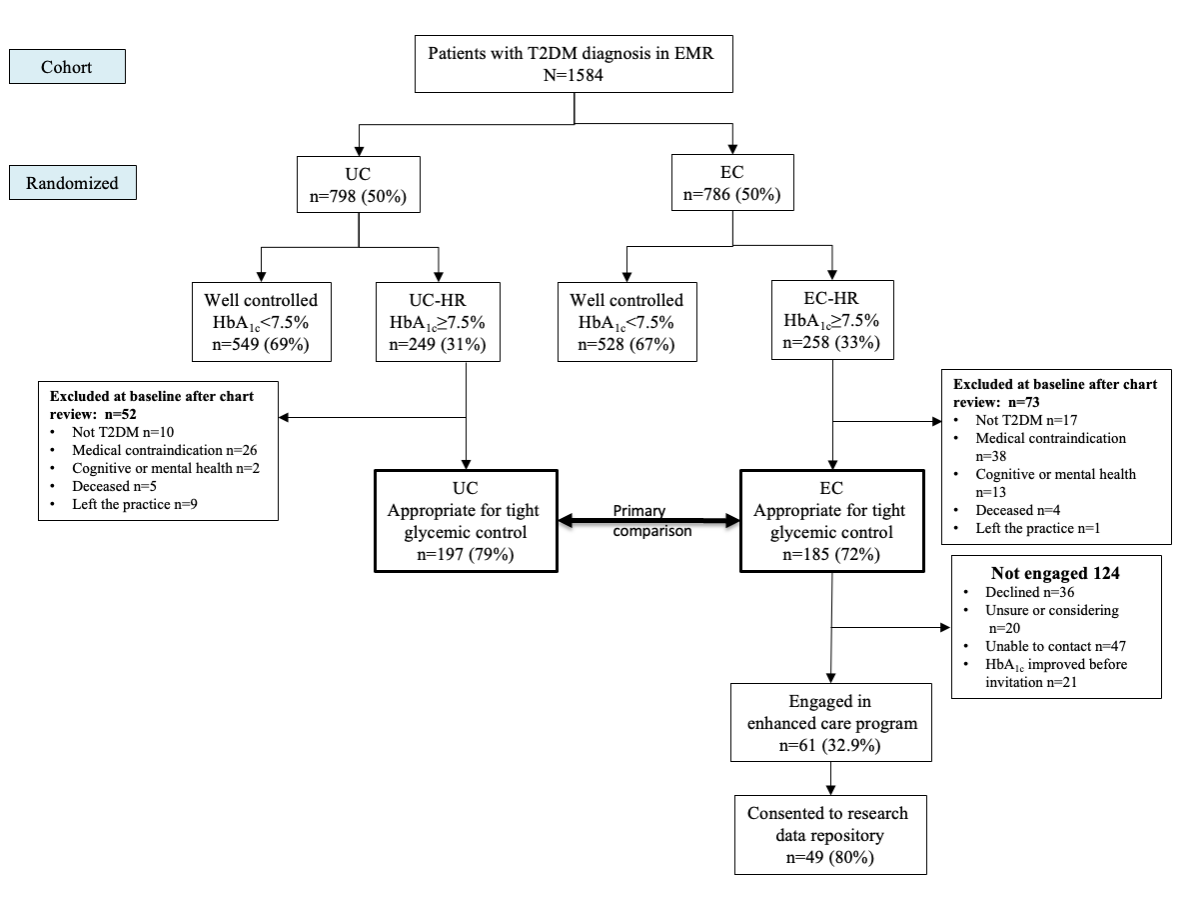
Patients with type 2 diabetes mellitus diagnosis (T2DM) in the electronic medical record (EMR). EC: enhanced care; HbA_1c_: hemoglobin A_1c_; HR: high risk; UC: usual care.

As shown in Figure 1, individuals within each study group were classified as UC-high risk (UC-HR) or EC-HR if they had a baseline HbA_1c_ level ≥7.5% and were medically eligible for improved glycemic control as determined by clinical review of the patients’ EHR data and discussion with a primary care physician (PCP), if necessary. Specifically, those individuals for whom tighter glycemic control (ie, HbA_1c_ <7.5%) was not recommended by the American Diabetes Association guidelines [25], older frail individuals at high risk of hypoglycemia and falls, those with a life expectancy of <6 months owing to a comorbid condition, or those with severe or untreated mental health conditions including eating disorders; women who were pregnant or breastfeeding; and those who had previous weight loss surgery were excluded from the high-risk cohorts.

### Recruitment

Several recruitment strategies were used to engage EC-HR individuals in the program. These included invitation by postal letters, EHR-based electronic messages, and phone calls by a program team member. In addition, for patients who did not respond to this outreach, an attempt was made to engage them face-to-face when they visited the clinic with their PCP or another health care provider.

### Program

#### UC Arm

Individuals within the UC arm received routine care during the 12-month study period. Routine care included PCP follow-up and the opportunity to receive nutrition counseling with a primary care–based dietitian who counseled patients to follow the standard calorie-restricted American Diabetes Association diet without a specific emphasis on dietary carbohydrate restriction [26].

#### EC Arm

All EC-HR individuals were invited to participate in the program (n=185). Of the 185 individuals, 61 (32.9%) of individuals engaged in the program and 80% (49/61) of individuals provided informed consent to participate in the data repository. Program participants used an intermittently viewed CGM (Abbott Freestyle Libre) and received personalized, low-carbohydrate dietary counseling from the program dietitian. In contrast to other CGM technologies including newer models of Freestyle Libre, the intermittently viewed CGM requires the user to scan the sensor to obtain results and does not have alarms for hypoglycemia or hyperglycemia. We use the term CGM rather than intermittently viewed CGM, as this is the overarching terminology used in consensus guidelines [1].

The sensor was placed by a dietitian and individuals had the opportunity to wear up to 7 CGMs during the 12-month program period. The program participants met one-on-one with the program dietitian. Although the dietitian was hired for program purposes, she did not have any advanced training that would preclude delivery of the intervention by dietitians in other primary care settings. During the first visit, the dietitian conducted a basic nutrition assessment (eg, diet recall) and instructed individuals on how to maintain an accurate food log that was used in conjunction with CGM data to facilitate nutrition coaching and tailored education.

After at least 10 days, the participants returned for a one-on-one visit with the dietitian who reviewed the CGM data and food logs and helped them understand how their dietary carbohydrate intake influenced their blood glucose levels. All the individuals were initially instructed to limit total daily dietary carbohydrate to ≤100 g per day, as this is a clinically relevant, pragmatic, and achievable target for many individuals [13,14]. The participants were subsequently advised to adjust their carbohydrate intake to optimize time in range, defined as blood glucose of 70-180 mg/dL (3.9-10 mmol/L). Specifically, the dietitian met with the patients to review the glucose monitoring data and food logs with the goal of discerning specific foods (eg, bread and bananas) that triggered glucose excursions. She then helped the participants to identify low-carbohydrate alternatives that met their dietary preferences and budget constraints and a lower carbohydrate target was specified (eg, a total of 50 carbohydrates per day) if the patients desired to count carbohydrates. When a lower carbohydrate goal was specified, the participants were instructed on how to count net carbohydrates (ie, total grams of carbohydrates–grams of fiber) to encourage intake of leafy greens and nonstarchy vegetables. In this way, nutrition counseling was tailored to the individual’s needs and aimed to accommodate differences in the degree to which individuals may need to restrict dietary carbohydrates to achieve glycemic control.

Although the dietary recommendations were drawn from many publicly available resources, the guiding principles of the program were based on the *Always Hungry* diet developed by Ludwig [27]. The dietitian also educated the patients regarding the potential risks and side effects of carbohydrate restriction, including hypoglycemia; hypotension; and physical symptoms of headache, fatigue, nausea, and constipation. Moreover, the dietitian communicated via the EHR with primary care clinical pharmacists or medical providers to facilitate timely changes in participants’ medications to avoid episodes of severe hypoglycemia and hypotension.

Multimedia Appendix 1 shows an example of the handouts used to teach the participants how to count carbohydrates. Multimedia Appendix 2 shows an example of the type of information reviewed during the visit with the dietitian.

#### Primary Outcome Measure

Baseline and follow-up HbA_1c_ levels were abstracted from the EHR. All the individuals had a baseline HbA_1c_ level obtained as part of routine clinical care before randomization. Follow-up HbA_1c_ data were obtained by the individuals’ PCPs as part of routine clinical care. To facilitate complete data availability, the program’s dietitian placed orders for HbA_1c_ levels for EC patients on PCPs’ behalf; PCPs could approve or cancel, as clinically indicated. The change in HbA_1c_ level was calculated by subtracting participants’ baseline HbA_1c_ level from the follow-up HbA_1c_ level.

#### Secondary Outcome Measure

Baseline and 12-month BMI were abstracted from the EHR. All the individuals had a baseline measurement of weight and height and calculated the BMI as part of routine clinical care before randomization. Follow-up BMI data were obtained by individuals’ PCPs as part of routine clinical care; we abstracted the follow-up BMI nearest to the end of the 12-month study period. Change in BMI was calculated by subtracting participants’ baseline BMI from follow-up values.

#### Exploratory and Process Outcomes

#### Program Engagement

We evaluated the rate of program engagement among the HR-EC cohort. We defined program engagement as the use of at least one CGM and having at least one meeting with the dietitian.

#### Change in Monthly Cost of Antihyperglycemic Medications

Change in the cost of antihyperglycemic medications was determined by subtracting the total cost of antihyperglycemic medications at baseline from the total cost of antihyperglycemic medications during follow-ups. Among the 61 individuals who engaged in the program, 49 (80%) consented to allow the study team members to review their complete EHR. Of the 49 participants, 1 (2%) participant left the practice, so 48 charts were reviewed for medication cost change data. A study team member reviewed the participants’ EHRs and documented the prescribed antihyperglycemic medications and dosages at the start and end of the study period. The cost of individual antihyperglycemic medications was determined using 2017 data from a private insurance claims database, Clinformatics DataMart Database (OptumInsight). Clinformatics DataMart Database contains deidentified claims capturing health care encounters (ie, office visits, outpatient visits, and inpatient visits) for >80 million privately insured adults and children. All the cost data in Clinformatics DataMart Database are standardized to enable comparisons of costs across patients, data sources, and geographic areas in a consistent manner. We also examined the change in monthly cost of antihyperglycemic medications for the subset of patients who were on insulin at baseline.

#### Change in CGM Metrics

CGM sensor metrics automatically calculated in the CGM report included average blood glucose level, percentage of time above range, percentage of time in range, percentage of time below range, and the number of low glucose events. In the subset of 45 patients in the EC-HR group who engaged in the program, consented data repository participation, and wore at least two sensors during the 12-month program period, we compared CGM sensor metrics using simple bivariate linear regressions with each CGM metric as the dependent variable and a dichotomous time variable to indicate first or last sensor.

#### Participant Experiences in the EC-HR Group

We conducted semistructured interviews by phone with participants from the EC-HR arm to explore their experiences with participating in the intervention [1]. A semistructured interview guide was selected so that consistent questions were asked across interviews and interviewers, while still allowing each interviewer to ask follow-up questions that were specific to each participant [28]. Phone interviews were selected to enhance participation among individuals who may have difficulty attending in-person. Interviews were conducted by team members trained in semistructured interviewing techniques, with regular feedback from a qualitative methodologist.

Those who agreed to share their data (49/61, 80% of participants in the EC-HR arm) were contacted by phone by the research coordinator and invited to participate. All participants were given up to 2 phone calls for recruitment and voicemails were left when available. Recruitment ended when sufficient interviews were completed to reach thematic saturation. The participants provided verbal consent. The phone interview was audio-recorded and then professionally transcribed.

### Statistical Analysis

Measures of central tendency (eg, proportions, means, and SDs) were used for all descriptive analyses. We compared changes in HbA_1c_ levels and BMI using an intention-to-treat difference-in-differences analytic approach using a linear mixed model. The difference-in-differences is the interaction term between a categorical variable denoting the period (eg, before vs after program period) and the study group (eg, UC-HR vs EC-HR). As a sensitivity analysis, we adjusted the models for age and gender. This had little effect on the parameter estimations for the difference-in-differences analysis. Therefore, we present only unadjusted results.

We estimated pre-post changes in HbA_1c_ levels and BMI among the 61 individuals who engaged in the program using a linear mixed model. We estimated pre-post changes in the cost of antihyperglycemic medications among the 79% (48/61) of individuals who consented to data repository participation. We calculated the mean pre-post costs and used paired *t* tests to determine the significance of difference. All analyses were conducted using STATA 16.0.

### Qualitative Data Analysis

A total of 3 team members (MD, TN, and CB) trained in qualitative analysis conducted an inductive thematic analysis [29,30]. First, we reviewed and organized all the transcripts using MaxQDA software. MD, TN, and CB independently coded the same transcript and discussed emerging ideas. We created a list of descriptive codes to represent meaningful segments of text; descriptive codes were then applied to the next transcript. We discussed the application of the codes to ensure that codes were being consistently applied by all team members. At this time, additional codes were added, and other codes were revised as needed to clarify the definitions. This process was repeated for the first 3 transcripts. Next, TN and CB independently coded the remaining transcripts. Application of the codes, relationships between the codes, and their meanings were discussed during regular team meetings. After coding, related codes were grouped into themes and summarized using structured summaries, including a narrative description of the theme and all the quotes associated with the theme.

## Results

### Baseline Characteristics

Demographic characteristics of the complete population (N=1584) were assessed at baseline (Table 1). Slightly less than half of the cohort was female (740/1584, 46.71%), and the mean age was 63.3 (SD 13.1) years. The UC and EC groups were similar at baseline. Among 185 EC-HR participants, 61 (32.9%) were engaged in the program. As shown in Figure 1, among the 124 individuals who did not engage in the study, the most common reasons for nonengagement included inability to contact individuals (47/124, 37.9%) and decline to participate (36/124, 29%).

**Table 1:** Baseline characteristics of all patients stratified by study group assignment.

Characteristic	All patients	UC^a^ group	EC^b^ group	UC-HR^c^ group	EC-HR^d^ group	Engaged in program
Population, n	1584	798	786	197	185	61
Age (years), mean (SD)	63.3 (13.1)	62.9 (12.8)	63.7 (13.4)	60.2 (11)	60 (12.2)	59 (11.9)
Women, n (%)	740 (46.71)	370 (46.4)	370 (47.1)	75 (38.1)	70 (37.8)	28 (46)
Baseline HbA_1c_^e^ level (%), mean (SD)	7.2 (1.5)	7.2 (1.5)	7.2 (1.6)	8.9 (1.4)	9 (1.6)	9 (1.4)
Baseline HbA_1c_ level (mmol/mol), mean (SD)	55.2 (16.4)	55.2 (16.4)	55.2 (17.5)	73.8 (15.3)	74.9 (17.5)	74.9 (15.3)
Baseline BMI (kg/m^2^), mean (SD)	34.6 (7.1)	34.8 (7.1)	34.3 (7.1)	35.5 (6.7)	35.8 (7.2)	37.3 (8.5)

^a^UC: usual care.

^b^EC: enhanced care.

^c^UC-HR: UC-high risk.

^d^EC-HR: EC-high risk.

^e^HbA_1c_: hemoglobin A_1c_.

### Primary Outcome

Baseline data were collected from all the patients. Of the 61 patients, 50 (82%) patients underwent at least one additional HbA_1c_ evaluation as part of routine clinical care during the 12-month study period. The mean time to follow-up HbA_1c_ level was 262 (SD 83) days. The HbA_1c_ level decreased by 0.41% (4.5 mmol/mol; *P*=.04) more from baseline to 12 months among EC-HR participants than among UC-HR participants. Adjusting for age and gender had little impact on the difference. In the pre–post comparison among the EC-HR participants who wore CGMs (n=61), HbA_1c_ level was 1.1% (12 mmol/mol) lower at 12 months compared with baseline (*P*<.001). The pre–post change in BMI in these participants was not statistically significant (within-group difference −0.6, *P*=.06; Table 2).

**Table 2:** Pre-post analysis^a^ for HbA_1c_^b^ and BMI of program participants at 12 months compared with baseline (N=61).

Characteristic	Baseline, mean (SEM^c^)	12-month, mean (SEM)	Difference within group at 12 months	*P* value (2-tailed test)
HbA_1c_ (%)	9 (0.11)	7.9 (0.12)	−1.1	<.01
HbA_1c_ (mmol/mol)	74.9 (1.2)	62.8 (1.3)	−12.1	<.01
BMI	37.3 (0.5)	36.7 (0.51)	−0.6	.06

^a^Values predicted from the mixed model not adjusting for age or gender.

^b^HbA_1c_: hemoglobin A_1c_.

^c^SEM: SE of the mean.

### Secondary Outcome

Baseline BMI was calculated for all the patients included in the cohort. Of the 61 patients, 53 (87%) patients had at least one additional BMI calculated as part of routine clinical care during the 12-month study period. The mean change in time to follow-up BMI was 287 (SD 81) days. There was no significant difference in BMI change from baseline to 12 months between the EC-HR and UC-HR participants (Table 3). Adjusting for age and gender had little impact on the difference. There was a trend toward modest weight loss among the EC-HR participants who wore CGMs (n=61) from baseline to 12 months, but this change was not statistically significant (−0.6 kg/m^2^; *P*=.06).

**Table 3:** Difference-in-differences analysis^a^ for HbA_1c_^b^ and BMI at 12 months compared with baseline [1].

Characteristic and group			Baseline, mean (SEM^c^)		12-month, mean (SEM)		Difference within group at 12 months		*P* value (2-tailed test)		Difference-in-differences			*P* value	
**HbA_1c_ (%)**												−0.41			*.*04
	UC-HR^d^	8.9 (0.11)		8.43 (0.12)		−0.47		.001							
	EC-HR^e^	9.01 (0.11)		8.12 (0.12)		−0.88		<.001							
**HbA_1c_ (mmol/mol)**												−4.5			.04
	UC-HR	73.8 (1.2)		68.6 (1.3)		−5.2		.001							
	EC-HR	75 (1.2)		65.2 (0.12)		−9.6		.001							
**BMI**												−0.11			.63
	UC-HR	35.45 (0.49)		34.89 (0.49)		−0.56		.001							
	EC-HR	35.84 (0.5)		35.17 (0.51)		−0.67		<.001							

^a^Values predicted from mixed model not adjusting for age or gender.

^b^HbA_1c_: hemoglobin A_1c_.

^c^SEM: SE of the mean.

^d^UC-HR: UC-high risk.

^e^EC-HR: EC-high risk.

### Exploratory and Process Outcomes

#### Change in Monthly Cost of Antihyperglycemic Medication

The average change in cost of antihyperglycemic medications from baseline to 12 months was –US $107 (SE of the mean 129.7; *P*=.41) among the 79% (48/61) of individuals who wore sensors and consented to data repository participation. Among the 52% (25/48) of individuals who used insulin, the average change in monthly cost of antihyperglycemic medications from baseline to 12 months was −$363 (SE of the mean 227.1; *P*=.12).

#### Change in CGM Sensor Metrics

Participants in the EC-HR group who engaged in the program and consented to data repository participation wore an average of 4.3 (SD 1.8) sensors during the 12-month program period. Of the 48 individuals, 4 (8%) individuals wore only 1 sensor. Of the 94% (45/48) of participants who wore at least two sensors, the average glucose decreased (−29.1 mg/dL, SD 9.4 mg/dL; *P*=.003), the average percentage of time above range decreased (−19%, SD 5.8%; *P*=.002), and the average percentage of time in range increased (17.7%, SD 5.4%; *P*=.002). The average percentage of time below range (+0.3%, SD 1%; *P*=.86) and the average number of low glucose events (+0.2 events, SD 1 event; *P*=.84) did not change significantly.

### Adverse Events

There were no major adverse events. Among the 61 participants, the most commonly reported events included skin irritation (6/61, 10%) or pain at the sensor site (3/61, 5%). Most endorsed transient symptoms did not preclude subsequent CGM use. Patients on oral anticoagulants noted bruising at the sensor site. Of the 61 participants, 1 (2%) patient reported an episode of sensor-detected hypoglycemia, which was determined to be owing to sensor error when she presented to the emergency department.

### Qualitative Results

#### Overview

Of the 61 participants in the EC-HR arm, 21 (34%) participated in semistructured interviews. Thematic analysis resulted in three themes related to EC-HR participants’ program engagement and CGM use: (1) ability to understand how specific foods impact blood glucose trends, (2) ease of following a low-carbohydrate diet, and (3) ease of blood glucose monitoring.

#### Ability to Understand How Specific Foods Impact Blood Glucose Trends

Participants in the EC-HR arm expressed that by using the CGM for blood glucose monitoring and reviewing their CGM data with the dietitian, they learned and better understood how different foods affected their blood glucose levels. Of the 21 participants, 1 (5%) participant explained:

The CGM was really good because it, it helped me just focus on, to see it in real time. I could watch my blood sugar rise, and then, see how high it rise, rose, after I ate somethin’ like that. So, it was really cool, I liked it.

Participant 010

Participants could easily relate the fluctuations in their glucose levels by looking at the spikes and dips in the graphs generated by the CGM. Many participants observed their glucose levels after eating food to understand how certain food items affect their glucose levels in real time. For example:

I really reflected on portion control with that study, because, I noticed—and you know like the types of food I was eating, because, it would jack my sugar my sugar way up or down based on what I was eating. Like a protein shake, even though it was, said it was low carb and all that, my sugar levels would raise and stay up for quite a while. Versus eatin’ like a boiled egg, you know, and a piece of toast. It would be more even. So, I learned like some foods are better for me than other foods are.

Participant 021

When reviewing the data independently and in collaboration with the dietitian, the participants could relate their mood, energy levels, concentration, and sleep cycles to the dietary choices they had made. Of the 21 participants, 1 (5%) participant summarized the helpful aspects of the intervention for them:

Workin’ with [the dietitian], and I think knowing, being able to see the spikes and stuff with what I ate. Oatmeal, I love oatmeal, but it spiked it [glucose level].

Participant 015

Finally, owing to their observations, participants described making different dietary choices in anticipation of the impact on their blood glucose. For example, participants reported exchanging foods with higher carbohydrates for those with more protein when their blood glucose levels were high. As 1 (5%) of the 21 participants explained:

I mean if it was too high, I would change what I was planning to eat next. So, if I checked it on my way home, and I was gonna pick up dinner, I might change, you know. I was gonna get pizza, but my sugar’s high, so instead I’m gonna get chicken.

Participant 008

When reviewing their CGM data in real time, the participants were able to respond and prevent subsequent spikes in their glucose levels.

#### Ease of Following a Low-Carbohydrate Diet

Most EC-HR participants reported that they were able to implement a low-carbohydrate diet as part of the study. They commented that they “do well on it,” “no problem,” or “it went pretty good.” However, some participants reported challenges in implementing and maintaining the diet during the intervention period, including preferences for high-carbohydrate foods, dealing with cravings, and the convenience of highly processed foods. Of the 21 participants, 1 (5%) participant who was able to reduce the number of carbohydrates consumed during the intervention still described the challenges:

[The diet was] not a lot of fun. Everything I love happens to be a carbohydrate...

Participant 013

Another participant described how difficult it was to find and prepare low-carbohydrate foods when busy or away from home, for example, while traveling for work:

I travel for work, so...being able to consistently find something to eat when I’m...getting, when it’s easier to go through fast food, than to find something lower carb, higher protein. Right. So that, you know, especially when my schedule’s, it’s halfway through my day and I end up being someplace I wasn’t expecting to be.

Participant 008

Though an uncommon experience, 5% (1/21) of the participants noted that feeling “different” or high-maintenance was a barrier to the low-carbohydrate diet during the intervention. They explained:

I’m tryin’ to hide [the diet]. You know there’s, uh, I, I don’t tell the whole family. My wife knows, my children know, my boys know, and...I basically don’t just pass it around because everybody’ll start treatin’ you different...“Oh can you eat this though? Can you eat this though?” You know, all that...they’ll make somethin’ for me that nobody else will eat. So, you know, I’d really rather not be treated like that.

Participant 002

As participants continued with the low-carbohydrate diet, many of them reported improvements in mood, concentration, energy, and sleep, and fewer cravings for carbohydrates. Participants described having “more energy when I’m not eatin’ carbs or drinkin’ caffeinated pop and all that” (Participant 001). However, these improvements were not universal. Some reported difficulty in concentrating and less energy, particularly in the first few days after starting the diet.

When asked about their intentions to maintain the low-carbohydrate diet in the future, more than half of the participants indicated that they would stick to the diet, whereas another 29% (6/21) of participants described making slight modifications. These participants often indicated that they had experienced improvements in their health and well-being while participating in the intervention. For example:

I actually am on a low carb diet [still], and I feel more energy ‘cause of the low carb diet.

Participant 003

Those who made modifications described being less strict with carbohydrate intake. Modifications included consuming more carbohydrates “in moderation” while still trying to “eat healthy” and “cut out junk.”

In contrast, 14% (3/21) of the participants reported not continuing to eat a low-carbohydrate diet after experiencing difficulty during the intervention. For example, 5% (1/21) of the participants, who described not reducing carbohydrates intake during the intervention, explained that they found it very restrictive to reduce carbohydrates:

That was hard because everything on it is stuff that I eat. I’m not a big fish eater, um...so it was hard, ‘cause I know with the carbs, you know, breads and pasta and...that’s the kind a stuff I like to eat...[During the intervention] I really didn’t change my diet out too much. I just ate less.

Participant 004

#### Ease of Blood Glucose Monitoring

The use of CGM influenced the lifestyle of the participants in many ways. First, participants described how using the CGM eliminated the need for a blood glucose monitor to check their blood glucose levels. For many participants, the CGM was more convenient:

It’s good for anybody to use anywhere at any time.

Participant 007

Furthermore, participants overwhelmingly preferred using the CGM to “poking my fingers all the time.” Participants were frequently irritated with pricking their finger for blood glucose checks because it was not only inconvenient but also painful:

The pain with the sticks. And I have short little fat fingers, and it’s really hard to grab those sticks and you know so much easier to put the monitor up there and...see my numbers.

Participant 006

Similarly, another participant commented that the CGM gives “instant results” instead of having to prick and squeeze the finger to measure the blood glucose.

Second, using the CGM resulted in more frequent blood glucose monitoring among EC-HR participants during the intervention. To illustrate, 5% (1/21) of the participants explained the frequency of their monitoring before the intervention:

I occasionally tested my blood sugar...It could go for...one every 3 months, to 2 months. Or was a hit and miss kind of a deal...

Participant 003

The participant went on to describe how this behavior changed during the intervention:

The changes that I noticed is that I checked my sugar more often. [Before the study] it could have been once every 3 months, once a week. So, there wasn’t a scheduled time, just whenever I...thought maybe I should. I would track it. And with the monitor it was right there, handy...It didn’t interfere with my life to check the blood sugars.

Participant 003

As described earlier, the convenience of the monitor made it easier for participants to incorporate blood glucose monitoring into their lifestyle. Another participant similarly described the laborious process when using the glucometer, rather than the “instant” and “handy” CGM system:

[With finger pricking] you got you know, the sensor, or the other thing you gotta, alcohol your finger, poke yourself, get the blood out of there. You know, check it. Alcohol your finger again, you know put everything away, throw away the strip. Uh, you know rather than just puttin’ the phone up to your arm, boom it’s done.

Participant 020

Third, using the CGM provided a more comprehensive picture of their blood glucose levels and blood glucose trends. For example, some participants described the benefit of being able to monitor trends in their blood glucose levels overnight and over time. Of the 21 participants, 1 (5%) participant explained how the additional knowledge about blood glucose trends influenced their diabetes management:

We found out also that the, uh...the blood glucose drops during the night, sometimes down in the 40s, which was [when I was] asleep. So, which is, you know, something you wouldn’t normally pick up. You can track it more, as to what’s been going on. It just gives lots more numbers.

Participant 017

Despite these benefits, several participants experienced barriers in using the CGM, including the challenges with the adhesive, skin infection, and the cost of CGM sensors postintervention. Most commonly, the adhesive on the CGM was insufficient, causing the sensor to fall off and then it has to be replaced during a subsequent visit. Although not a common experience, 5% (1/21) of the participants complained of skin infections at the site of the sensor insertion. Finally, some participants expressed that they would like to continue using the CGMs to monitor their blood glucose following the intervention, but were limited by the cost and their insurance:

Rather than poking my finger, I totally would go for that [the CGM] in place of the other [glucometer]. But my insurance won’t cover it, so I can’t continue on.

Participant 004

## Discussion

### Principal Findings

This 12-month pragmatic QI program evaluation examined whether CGM technology—in conjunction with low-carbohydrate nutrition counseling—could reduce HbA_1c_ among patients with suboptimally controlled T2DM. The results show a significantly greater reduction in HbA_1c_ (–0.41%; –4.5 mmol/mol; *P*=.04) among individuals randomized to EC (n=185) compared with individuals randomized to UC (n=197). The improvement in HbA_1c_ level was not accompanied by an increase in the cost of antihyperglycemic medications and was associated with improved CGM metrics. Qualitative results indicated that EC-HR participants positively viewed engagement with the intervention and use of the CGM; however, experiences with the low-carbohydrate diet were more variable.

A recent systematic review and meta-analysis revealed significant improvements in glycemic control at 3 and 6 months among low-carbohydrate diet group participants, but these improvements diminished by 12 months [1]. In contrast, other studies testing the effectiveness of very low-carbohydrate ketogenic diets demonstrate significant and durable improvements in HbA_1c_ levels up to 24 months [2,3]. In this study, although only 32.9% (61/185) of EC participants engaged in the program, we observed a significant reduction in HbA_1c_ at 12 months. Given that this program used low-intensity diet counseling, our results suggest that CGM technology may facilitate adherence to a low-carbohydrate diet.

CGM technology is rapidly advancing and is increasingly recognized as a novel tool to support personalized T2DM management [21]. However, to date, few strategies have examined the role of CGM technology in supporting T2DM management among patients in general practice settings [4]. Among existing interventions targeting individuals with suboptimal glycemic control, many aim to promote medication intensification and adherence to prescribed regimens [4,31]. In contrast to these existing strategies, CGM technology coupled with personalized nutrition counseling may facilitate improved glycemic control through behavior change without medication intensification. A small, nonrandomized study used CGM technology and personalized nutrition counseling to promote a low glycemic index breakfast and demonstrated a reduction in glycemic variability at 2-week follow-up [32]. In this study, we similarly observed improved glycemic control without an increase in antihyperglycemic medication use.

Previous studies have demonstrated the efficacy of low-carbohydrate diets for T2DM management [13,33]. However, few previous studies have demonstrated the effectiveness of low-carbohydrate diets among individuals in real-world settings [34,35]. A primary care–based intervention offered an in-person low-carbohydrate program to a nonrandomized group of patients and demonstrated significant improvements in weight and HbA_1c_ levels over the 13-month study period [34]. A web-based intervention available to the general public similarly demonstrated reductions in HbA_1c_ levels and body weight among a nonrandomized cohort of program completers [35]. We augment these previous findings by demonstrating a significant reduction in HbA_1c_ levels among a randomized cohort of patients using an intention-to-treat analytic approach. Our data suggest that personalized nutrition counseling focused on dietary carbohydrate restriction and guided by individuals’ CGM data are more effective than routine PCP and dietitian follow-up among patients with suboptimally controlled T2DM. Intervention participants positively engaged in the intervention, noting the benefits of knowledge and support provided by the dietitian. Although not all participants maintained a low-carbohydrate diet after the intervention, they were able to successfully reduce carbohydrate intake during the intervention and noted improvements in their physical health and well-being.

Among the 185 EC-HR individuals eligible for tight glycemic control, 61 (32.9%) engaged in the program. This is considered a high rate of participation, given that individuals were randomized before the program invitation and all eligible individuals were included in the denominator. Among the 124 individuals who did not engage in the study, the most common reason for nonengagement was the inability to contact eligible individuals (47/124, 37.9%), whereas 29% (36/124) declined to participate in the program and 16.1% (20/124) were uncertain about participation and did not engage during the study period. We did not explore reasons why individuals declined to participate and this is something that could be investigated in future work. To our knowledge, few previous studies have explored barriers to and facilitators of CGM technology as a diabetes self-management tool among patients with T2DM [36,37]. Although CGM technology can empower and motivate patients with type 1 diabetes mellitus who are accustomed to routine self-monitoring of blood glucose to guide insulin dosing [36], CGM technology may enhance diabetes-related distress, which is a known barrier to T2DM self-management [38]. Moreover, carbohydrate-restricted diets may not appeal to some patients’ preferred eating patterns, thus underscoring the need for additional personalized nutrition approaches [39,40].

Over the 12-month study period, we observed statistically significant within-group changes in HbA_1c_ levels and BMI among UC-HR and EC-HR individuals. This may reflect contemporaneous changes in clinical practice with a shift away from using obesogenic antihyperglycemic medications (eg, insulin and sulfonylureas) and toward newer agents that may facilitate weight loss and patient compliance (eg, sodium-glucose cotransporter-2 inhibitors and glucagon-like peptide 1 receptor agonists). Consistent with results of previous literature on primary care–based interventions to improve glycemic control among patients with suboptimally controlled T2DM [4], we did not observe a significant between-group change in BMI.

### Limitations

First, we recruited individuals from a primary care clinic within a US academic medical center and the program content was delivered by a single dietician; therefore, our results may not be generalizable to other clinics or populations. Second, we did not evaluate outcomes beyond 12 months and were therefore unable to assess long-term changes in glycemic control. Third, as this was a pragmatic QI study that did not require consent for randomization, we used HbA_1c_ levels and BMI data that were obtained for clinical purposes. Therefore, there was variability in the time between the baseline and follow-up assessments. Moreover, we were limited in the type of data we could abstract from the EHR and we did not have the ability to review changes in antihyperglycemic medication use for the complete cohort. Among the EC-HR subset of individuals who provided written informed consent for EHR review, medication intensification did not drive between-group change in HbA_1c_ levels. Follow-up interviews were limited to the EC-HR data repository participants and the experiences of participants in the UC arm were not explored. Finally, we cannot discern the comparative effectiveness of dietary carbohydrate restriction plus CGM versus dietary carbohydrate restriction or CGM alone. However, our results suggest that CGM may facilitate the translation of low-carbohydrate diets into routine clinical practice. This contrasts with previous efficacy and effectiveness studies of dietary carbohydrate restriction, which are often intensive or require study-specific personnel, which may limit their generalizability to routine practice settings.

### Conclusions

Many patients with T2DM do not achieve optimal glycemic control [7] despite clinical practice guideline recommendations [15,16] and interventions to support the intensification of patients’ antihyperglycemic regimens [4]. There is now growing evidence to support the use of carbohydrate-restricted diets to help patients reduce blood glucose levels and medication use [16,18,41]. However, the degree to which individual patients need to restrict dietary carbohydrates to achieve benefits is unknown [19]. Our findings demonstrate that the use of CGM technology and personalized nutrition counseling focused on dietary carbohydrate restriction can help patients with suboptimally controlled T2DM to improve HbA_1c_ levels without increasing antihyperglycemic medication use. As CGM technology evolves [21] and carbohydrate restriction is increasingly accepted as a powerful tool to support T2DM self-management, this program may be a scalable and sustainable strategy to help and empower patients with T2DM to achieve glycemic control.

## Appendix

Multimedia Appendix 1 Method to calculate net carbohydrates.

Multimedia Appendix 2 An example of the nutrition counseling template used by the program dietitian.

## Abbreviations

CGM: continuous glucose monitoring
EC: enhanced care
EC-HR: enhanced care-high risk
EHR: electronic health record
HbA_1c_: hemoglobin A_1c_
IRB: institutional review board
PCP: primary care physician
QI: quality improvement
T2DM: type 2 diabetes mellitus
UC: usual care
UC-HR: usual care-high risk
