# Supplementary material for: Continuous Glucose Monitoring With Low-Carbohydrate Nutritional Coaching to Improve Type 2 Diabetes Control: Randomized Quality Improvement Program
Source: J Med Internet Res. 2022 Feb 2;24(2):e31184. doi: 10.2196/31184 (PMC8851329; doi:10.2196/31184)

**Appendix 1: How to Calculate Net Carbohydrates**

**Black Beans**


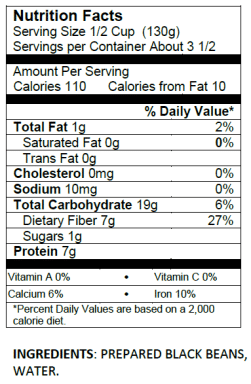


So for ½ cup beans:

19g total carbs

– 7g fiber

= 12g net carbs

**3. Subtract grams of fiber**

**= Net carbs**

**2. Check total grams of carbohydrate**

**1. Check the serving size**


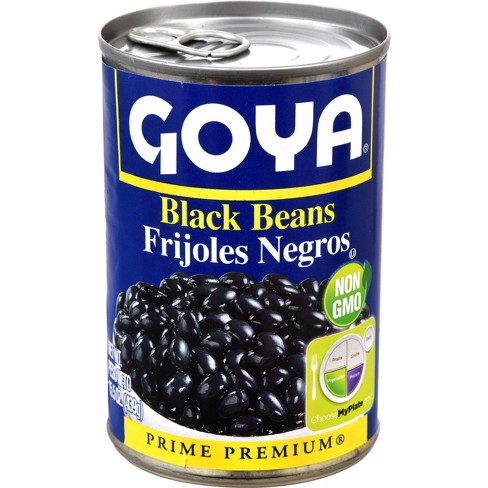


**Small Tortillas**

**
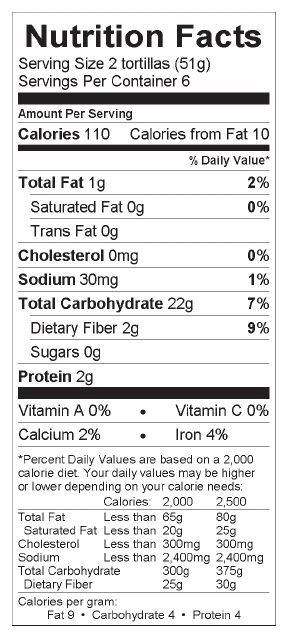
**


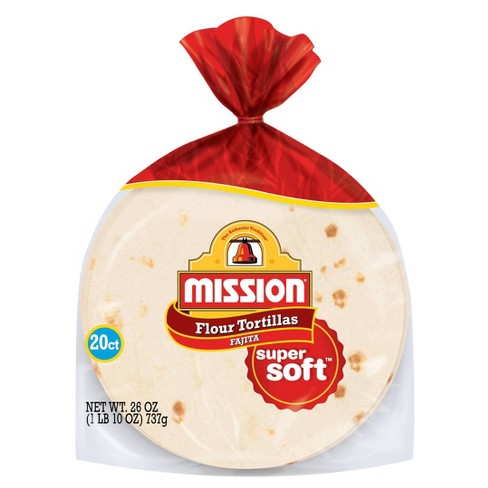

Supplement: Multimedia Appendix 1 [file jmir_v24i2e31184_app1.docx]
