# Supplementary material for: Continuous Glucose Monitoring With Low-Carbohydrate Nutritional Coaching to Improve Type 2 Diabetes Control: Randomized Quality Improvement Program
Source: J Med Internet Res. 2022 Feb 2;24(2):e31184. doi: 10.2196/31184 (PMC8851329; doi:10.2196/31184)
Supplement: Multimedia Appendix 2 [file jmir_v24i2e31184_app2.docx]

**Appendix 2: An example of the nutrition counseling template used by the program dietitian.**

The dietitian entered patient-specific information to the sections marked with “***”.

*Continuous Glucose Monitoring*

The patient's CGM data was uploaded and the PDF report was reviewed with the patient alongside the patient’s food, energy, and cravings logs.

We identified patterns where blood glucose out of range. These included ***

A new CGM sensor (#***) was placed on ***DATE*** and will end on *** (10 days later)

*Nutrition Education and Counseling*

- We discussed the relationship between carbohydrate intake, blood glucose, and insulin.
- We identified 3 main ways that blood glucose can be lowered: food, medication, exercise.
- We discussed the key sources of carbohydrate in the diet: fruits and juice, starchy vegetables and legumes, grains, dairy, and pure sugar (soda, desserts, candy).
- I recommended the goal of gradually reducing carbohydrate intake to <100g net carb per day to improve blood glucose control.
- I recommended starting by choosing a lower-carbohydrate breakfast such as eggs in place of breakfast cereal or toast.
- We discussed carbohydrate counseling using nutrition labels (see below).

The patient was given a symptom log and shown how to record any potential adverse effects of a low carbohydrate diet (e.g. Muscle cramps, headache, fatigue). The patient was instructed to contact the RD with any questions or if symptoms persisted.
